# Synthesis of heavy hydrocarbons at the core-mantle boundary

**DOI:** 10.1038/srep18382

**Published:** 2015-12-17

**Authors:** Anatoly B. Belonoshko, Timofiy Lukinov, Anders Rosengren, Taras Bryk, Konstantin D. Litasov

**Affiliations:** 1 Condensed Matter Theory, Theoretical Physics, AlbaNova University Center, KTH Royal Institute of Technology, 106 91 Stockholm, Sweden; 2 Center for Quantum Materials, Nordita, Roslagstullsbacken 21, AlbaNova University Center, SE-106 91 Stockholm, Sweden; 3 Institute for Condensed Matter Physics of the National Academy of Sciences of Ukraine, 1 Svientsitskii Street, UA-79011 Lviv, Ukraine; 4 V. S. Sobolev Institute of Geology and Mineralogy, SB RAS, Novosibirsk, 630090, Russia; 5 Novosibirsk State University, Novosibirsk, 630090, Russia

## Abstract

The synthesis of complex organic molecules with C-C bonds is possible under conditions of reduced activity of oxygen. We have found performing *ab initio* molecular dynamics simulations of the C-O-H-Fe system that such conditions exist at the core-mantle boundary (CMB). H_2_O and CO_2_ delivered to the CMB by subducting slabs provide a source for hydrogen and carbon. The mixture of H_2_O and CO_2_ subjected to high pressure (130 GPa) and temperature (4000 to 4500 K) does not lead to synthesis of complex hydrocarbons. However, when Fe is added to the system, C-C bonds emerge. It means that oil might be a more abundant mineral than previously thought.

The subject of the origin of organic materials on the Earth is multi- and cross- disciplinary, and is central to such topics as abiotic oil genesis, the origin of life, CO_2_ sequestration, and mantle convection^1^,^2^,^3^,^4^,^5^,^6^,^7^,^8^,^9^,^10^,^11^,^12^,^13^,^14^,^15^,^16^. Quite a few experiments^1^,^4^,^5^,^17^ and computations^18^,^19^ have been conducted and theories^2^,^3^,^15^,^16^,^20^ formulated to explain the origin of organic material. The common feature of all these theories is that they require a number of favourable circumstances to provide the conditions for chemical reactions that would result in hydrocarbons. All experiments and computations have been concentrated on methane and did not consider a chemical composition more natural to the Earth interior. Hydrocarbons consist of C and H atoms. These elements are present in water and carbon dioxide as well as in a number of minerals (e.g. Mg(OH)_2_ and MgCO_3_). The C, O, and H containing rocks can be dragged by subduction to the core-mantle boundary, which is considered as a final destination graveyard of some oceanic plates. Oxidized hydrogen and carbon species can be delivered by deep-sinking subducting slabs, whereas an alternative source of carbon and hydrogen would be the core itself^10^.

In this study we have attempted to answer what happens to a H_2_O-CO_2_ (C-O-H) mixture subjected to high pressure (P) and temperature (T) in contact with rocks. Several studies point to the possibility of CH_4_ formation in the upper mantle. Indeed, flow of methane of abiotic origin is well documented^21^. High P-T experiments have demonstrated the transition of CH_4_ into heavy hydrocarbons in the absence of oxygen^5^. Whether the C-C bond characteristic to heavy hydrocarbons will emerge in a H_2_O-CO_2_ mixture under the conditions of deep Earth interior remains unknown. Such a bond, if formed, would indicate a possibility of endogenic oil synthesis. Such possibilities are debated at present and a number of scenarios have been put forward^3^,^16^.

To study the water-carbon dioxide mixture we applied the *ab initio* molecular dynamics method (AIMD, see Method). This method is perfectly suited to study systems with chemical reactions^22^,^23^ unlike a classical approach where the chemical identity of the components remains unchanged. The method has been applied before to study methane^19^,^20^, H_2_O^24^ and CO_2_^25^ at high PT and the reliability of the method is well established. To make sure that the method is capable of providing results in close agreement with experiment and that our set up is valid, we computed the structure of H_2_O (Supplemental Materials) that turned out to be in close agreement with the most reliable experiments^26^. Since the reliability of the Method improves on increasing of pressure and temperature (Supplemental Materials) we consider the obtained results reliable.

The conditions of the most relevant runs are summarized in Table 1. We paid particular attention to two obstacles. One is metastability of the obtained state and another is contact of the C-O-H system with iron. In order to avoid the metastability all runs were performed starting from atomic and molecular mixture of atoms. Also, the impact of size was explored when simulating atomic C-O-H and molecular mixture of H_2_O-CO_2_. Neither size nor initial structure affect the final result and similar structures of the final product are obtained in all cases of similar composition. It was observed that molecules of water as well as carbon dioxide readily dissociate in the presence of iron. Figure 1 shows initial and intermediate snapshots of H_2_O-CO_2_-Fe. By comparing the two snapshots we see that molecules dissociate and atoms diffuse to significant distances (note that the atoms in Fig. 1 are connected not because they form a bond – the connecting bars simply show how far the neighbours moved apart). Therefore, one can conclude that the resulting substance is an atomic liquid mixture. Very long runs, up to 150,000 time steps, have been performed to ensure an equilibrium state. Radial distribution functions (RDF) were computed for all systems (Figs 2 and 3). From Fig. 2 we see that the C-C and C-H bonds develop as soon as Fe is added to the system. The C-C bond is clearly formed in the presence of Fe because the RDF in Fig. 2 demonstrates a narrow tall peak at the distance of the C-C bond. This is a typical feature of a chemical bond (see, for example the RDF for O-H in water in Fig. S1 where the first peak is due to the chemical bond between O and H). The bond is formed because the oxygen becomes bonded to Fe instead of C (the C-O peak disappears). That is, under the conditions of the core-mantle boundary the Fe affinity to O is stronger than the affinity of C to O. Similarly, the C-H peak is exactly at the distance where the C-H bond forms (Fig. 2). The C-H RDF peak is less pronounced than the C-O peak because of the weaker bond but also because of the low atomic weight of the H atom. At the temperature 4500 K the H atom is very mobile. While formation of abiotic methane in nature is known, for the first time we demonstrate the synthesis of hydrocarbons with a C-C bond under conditions that are typical of the Earth core-mantle boundary. The C-O RDF peak vanishes indicating the reduced conditions. C and O demonstrate strong affinity to Fe atoms, while Fe-H correlation is weak (Fig. 3). We want to emphasize that the formation of C-C bond is not due to affinity of C to Fe. The first peak of Fe-C RDF is wide and broad indicating that there is no chemical bond between Fe and C. On the contrary, the first peak of C-C RDF (Fig. 2) is narrow and high, a typical feature of a chemical bond. The integration of C-C RDF provides us with the number of C that is bonded to C atoms which is close to 1. This suggests that most of C atoms form C-C bond and a typical C-H molecule has two C atoms. We note that despite that we performed extraordinary long calculations with a number of atoms that is large by the standards of ab initio molecular dynamics, it might still be insufficient to fully capture the formation of larger C-H molecules. The affinity of C and O to Fe suggests that C and O can be light components of the Earth core. Therefore, our simulations support the relatively high amount of oxygen^27^ in the outer core. While the authors of Ref. 27 relate the core oxidation state to the oxygen content in the magma ocean, it is likely that the core current state is significantly affected by the mass exchange and chemical reactions at the border of core and mantle as well as at the inner-outer core boundary.

It is important to discuss possible migration mechanisms and survivability of hydrocarbons, presumable formed at the core-mantle boundary, in the mantle plumes or upwelling mantle. The arguments below indicate that successful delivery of hydrocarbons from the core-mantle boundary to sub-lithospheric depths is possible.

The mantle plumes or convection upwelling originated at the core-mantle boundary are explained by the necessity to release heat from the core accumulated due to poor thermal conductivity of the lower mantle. Since most silicates of the lower mantle are highly refractory, ‘violent’ plume ascent is only possible with an additional fusible component. Thus, plumes from the core-mantle boundary are thought to have thermo-chemical nature^28^. The most likely candidates for fusible chemicals in the mantle plumes are alkali-bearing species, C-O-H volatiles, and carbonates.

An important requirement for plume motion would be stress-induced melting and dissolution-precipitation of the fusible component at the front and rear of the plume, respectively^29^. For this process one would have a volatile-bearing melt with low solubility of silicates (ca. 5–15%, but not zero) at the temperature of lower mantle geotherm (or slightly higher). The possible candidates are alkali-bearing silicate melt, hydrous silicate melt, carbonatite melt, and hydrocarbon-bearing melt. Alkaline silicate melt and hydrous silicate melt cannot be considered since a huge amount of silicate can be dissolved in these melts and the process of plume ascent will be easily terminated by progressive reactions with the surrounding silicate matrix. Carbonated or carbonatite melt is a likely candidate, but it cannot survive through the lower mantle due to reduction to diamond or other carbon-bearing species (carbide) if we assume redox state of the lower mantle close to the Iron-wustite (IW) buffer^30^,^31^. Thus, hydrocarbon-bearing or hydrous hydrocarbon-bearing melt might be the best candidate for the liquid portion of a mantle plume arising from the core-mantle boundary (Fig. 4).

There is a limited amount of information about hydrocarbon phase relations and reactions with silicates in the lower mantle due to an extremely difficult experimental setup. The data for melting of volatile-bearing peridotite in the system buffered by the IW buffer at 1–3 GPa indicated negligible solubility of silicates in coexisting CH_4_-H_2_O fluid^32^. However, recent melting experiments on peridotite and eclogite systems with reduced C-O-H fluid at 3–16 GPa indicated a significant solubility of silicates in the coexisting C-O-H fluid. The diamond or graphite traps contained abundant microinclusions of silicates after experiments^33^. The composition of the fluid was not measured in the experiments, whereas theoretical estimates indicate a mixture of H_2_O with methane and possibly heavier hydrocarbons. A similar fluid/melt containing H_2_O and hydrocarbons with a relatively low solubility of silicate components along the mantle geotherm can exist through the lower mantle and can be considered as the most reliable candidate for the fusible component of mantle plumes from CMB.

Concluding, we demonstrated that formation of a hydrocarbon mixture is highly probable under reducing conditions at the core-mantle boundary. Such a formation might contribute to the explanation of abiotic oil formation, mechanism of ascending hot plumes, ultra-low velocity zone at the CMB, and amount of oxygen in the liquid outer core.

## Method

**Figure 1:**
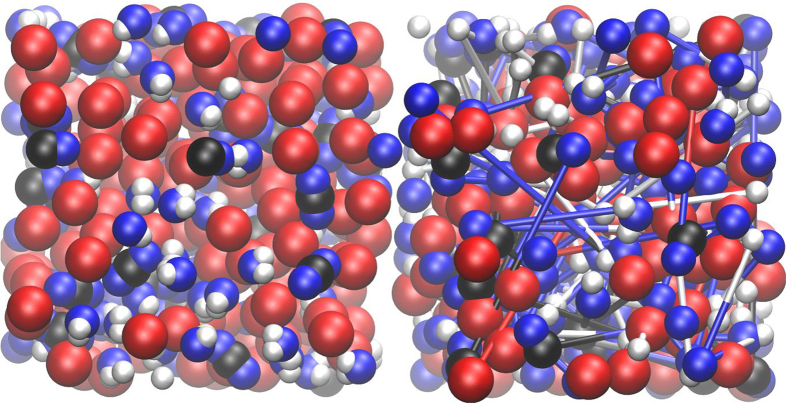
The projection of atomic coordinates of the simulated H_2_O-CO_2_-Fe system (Table 1) on the XY plane for two time steps. Interatomic bonds calculated during the first step (step 1, 0 ps, left) are shown on the final step picture (step 32590, 8 ps, right). The simulation was conducted in a cubic box with side 13.92 Å. The colors of atoms correspond to the elements: ○ hydrogen (160 atoms), 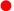 iron (136 atoms), 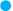 oxygen (160 atoms) and ● carbon (40 atoms), totally 496 atoms. Animation of the first 4000 timesteps of this simulation provided in Supplemental materials.

We studied C-O-H, C-O-H-Fe, H_2_O-CO_2_, and H_2_O-CO_2_-Fe systems (Table 1) by means of *ab initio* molecular dynamics simulations where energies and forces have been calculated using density functional theory as implemented within the Vienna Ab Initio Simulation Package (VASP 5.3)^34^,^35^,^36^. The plane wave cut-off was chosen as the largest for the involved elements (C, O, H, Fe) and in addition it was increased by 20 percent considering high pressure, so the cut-off was 480 eV. All simulations have been performed with gamma point only since the systems are very large by AIMD standards. The PBE (Perdew-Burke-Ernzerhof) exchange correlation functional^37^ was used in all simulations. The time step ∆t was equal to 0.25 femtosecond (fs) because of high PT conditions and low atomic weight of the involved elements, in particular hydrogen. The number of steps was at least 80,000 to get a time of observation of at least 20 picoseconds. The error was calculated using the blocking technique. Most of the working runs were performed in the NVT ensemble using the Nosè thermostat to control the temperature. The initial configurations in all cases were created by first generating a liquid structure of Ar in a box well above the melting T. Then, all Ar atoms were substituted by either C, O, H, or Fe atoms or an H_2_O or CO_2_ molecules (or a vacancy). We know that a system can remain metastable in the molecular state because breaking chemical bonds requires considerable energy. We tried monatomic C-O-H and C-O-H-Fe systems as well as molecular H_2_O-CO_2_ and H_2_O-CO_2_-Fe systems. The size of the computational box was adjusted to match approximately the pressure in the vicinity of the core-mantle boundary (CMB). All the details of studied cases are provided in Table 1. After a long equilibration period of at least 60,000 time steps the data was accumulated and averaged over at least 5 ps. We checked the averages by calculating them over smaller periods of time and the statistical error is negligible for our purposes (statistical errors in pressure are less than 1 GPa and errors in temperature are smaller than 6 degrees). The structural data (RDF) was computed for each pair of atoms as described in Ref. 38. The tools we used were tested by computing the structure of liquid H_2_O at ambient pressure at the melting temperature of the *ab initio* model and nearly perfect agreement with the most reliable experimental structural data^26^ was obtained (Supplemental Materials Fig. 1S).

## Additional Information

**How to cite this article**: Belonoshko, A. B. *et al.* Synthesis of heavy hydrocarbons at the core-mantle boundary. *Sci. Rep.* **5**, 18382; doi: 10.1038/srep18382 (2015).

## Supplementary Material

Supplementary Information

Supplementary Video

## Figures and Tables

**Figure 2:**
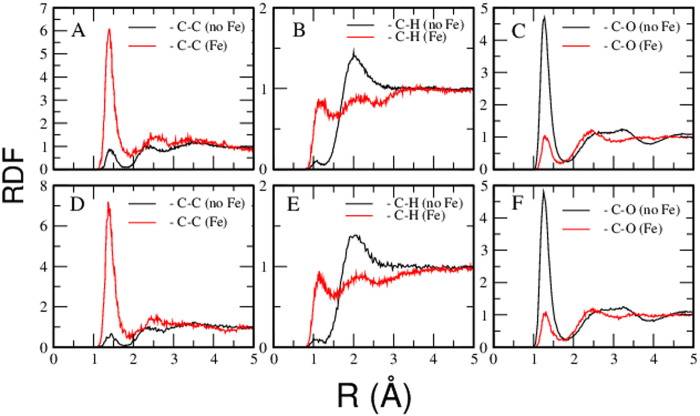
Structure of the Fe-C-O-H system under conditions as indicated in Table 1. The upper row provides results of simulation obtained for the atomic mixtures of elements; the bottom row are results for the initially molecular mixture of water and carbon dioxide. Each part provides a comparison of a partial RDF obtained in the system with and without Fe. The results for the different initial structures are nearly identical that suggests that water and carbon dioxide become an atomic mixture at the conditions at the core mantle boundary. Figure A,D show the appearance of a C-C bond with a length typical in organic compounds^39^ around 1.5 Å as the C-O-H system is brought in contact with Fe. Figure B and E show the appearance of the C-H bond with a typical length of around 1.1 Å, also typical of organic compounds^27^. Strong C-O (Figures C and D) bonds present in the system without Fe becomes almost non-existent in the system with^40^,^41^,^42^,^43^,^44^ iron.

**Figure 3:**
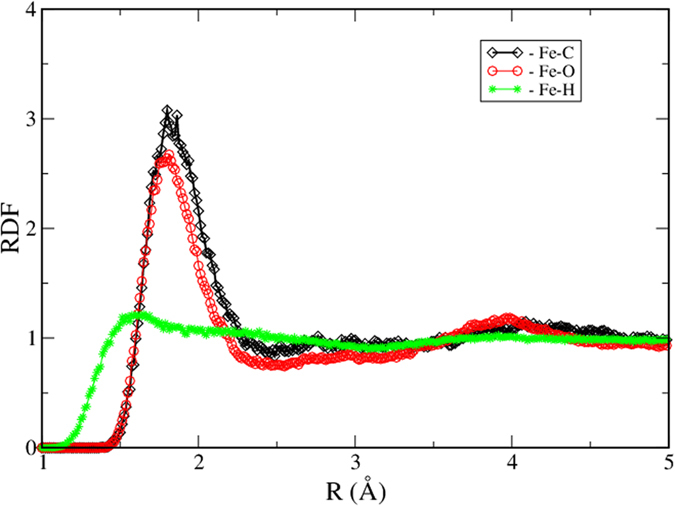
Radial distribution function of Fe and light elements. The affinity of Fe to C and O suggests that C and O elements will be transported to the outer core while H is less likely to be present in the core.

**Figure 4:**
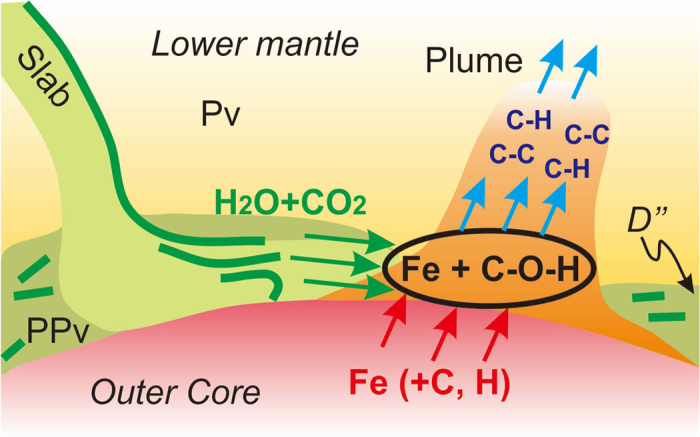
Formation of unsaturated hydrocarbons marked as C-C and C-H bonds at the core-mantle boundary via reaction of C-O-H-bearing fluid/melt from subducting slab with Fe from the core. An alternative source for C and H would be the metallic core itself. Green slices represent former oceanic crust. Pv – perovskite, PPv – post-perovskite-bearing layers in colder zones of the CMB.

**Table 1:** Simulated systems of C-O-H-Fe composition.

System	C	O	H	Fe	L, Å	P, GPa	T, K
C-O-H	40	160	160	0	11.28	138.8	4500
C-O-H-Fe	40	160	160	136	13.68	137.3	4500
H_2_O-CO_2_	86	342	340	0	14.64	128.9	4500
H_2_O-CO_2_-Fe	40	160	160	136	13.92	110.8	4500
