# Supplementary material for: Synthesis of heavy hydrocarbons at the core-mantle boundary
Source: Sci Rep. 2015 Dec 17;5:18382. doi: 10.1038/srep18382 (PMC4682099; doi:10.1038/srep18382)
Supplement: Supplementary Information [file srep18382-s1.pdf]

Supplementary Materials

Synthesis of heavy hydrocarbons at the core-mantle boundary

Anatoly B. Belonoshko[1,*], Timofiy Lukinov[1], Anders Rosengren[1,2], Taras Bryk[3] & Konstantin D. Litasov[4,5]

[1]Condensed Matter Theory, Theoretical Physics, AlbaNova University Center, KTH Royal Institute of Technology, 106 91 Stockholm, Sweden

[2]Center for Quantum Materials, Nordita, Roslagstullsbacken 21, AlbaNova University Center, SE-106 91 Stockholm, Sweden

[3]Institute for Condensed Matter Physics of the National Academy of Sciences of Ukraine, 1 Svientsitskii Street, UA-79011 Lviv, Ukraine

[4]V. S. Sobolev Institute of Geology and Mineralogy, SB RAS, Novosibirsk, 630090, Russia

[5] Novosibirsk State University, Novosibirsk, 630090, Russia

** To whom correspondence should be addressed. Email: anatoly@kth.se.*

Figure S1. (Supplemental Data)

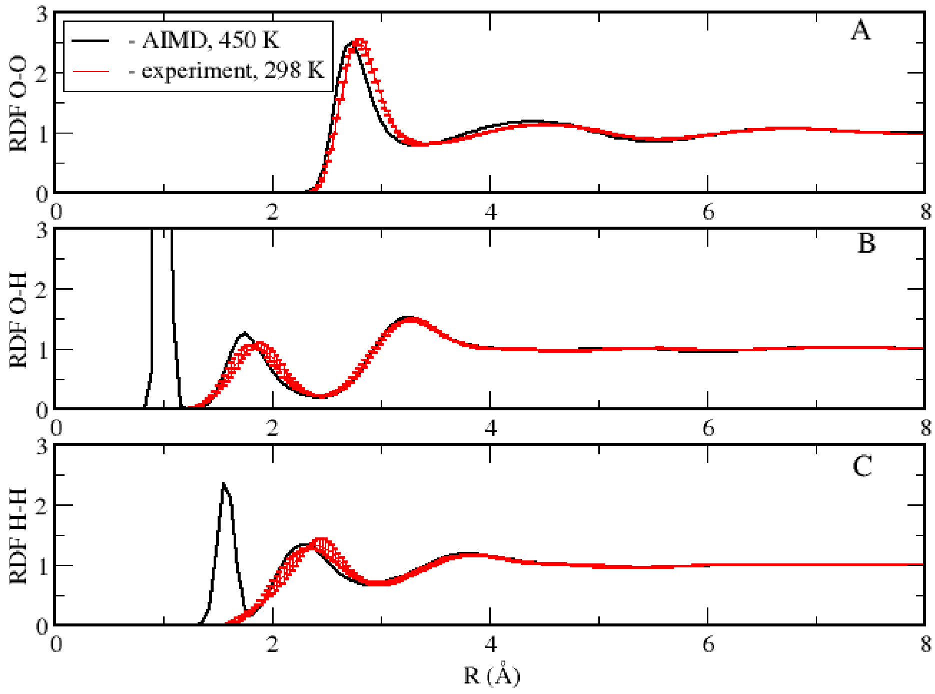

S1. Computed structure of water at the melting point (determined using the two-phase method[40.41]) and ambient pressure compared to experiment[26] at T=298 K. The H_2O ice melts at 450 K according to simulations (this is due to neglect of van der Waals forces and zero-point motion[24], both of these can be neglected at high pressure and temperature). Note, that comparison of DFT-PBE water and real water at the room temperature provides the impression that the DFT-PBE provides too high and sharp peaks in comparison to experiment. This is simply because DFT-PBE water at 300 K and 1 bar is metastable and supercooled – the melting T of the DFT-PBE water is around 450 K as we obtained. Only when we compare both waters at their melting point – that is in their corresponding field of stability – we get good agreement. In addition to

the excellent agreement of the experimental and calculated RDFs, we also obtain a self-diffusion coefficient of about 2.3 cm^2/sec which is in very good agreement with experiment. At the same time, simulations of water with the same approach at ambient temperature lead to overstructuring and very low self-diffusion[43,44]. Our simulations of water allow to quantify the errors of DFT-PBE and it is about 150 K. This is clearly negligible in comparison to 4500 K that is the temperature at the core-mantle boundary. The first peak of the calculated O-H and H-H RDF is due to the intra-molecular O-H and H-H bonds. These bonds do not show up in experimental spectra. The agreement is very good, almost within the experimental error bars. Since the precision of the method improves with increase of temperature (zero point motion becomes less important) and pressure (dispersion becomes increasingly weaker in comparison to repulsive forces), we can rely on this method in our simulations performed under the extreme PT conditions at the core-mantle boundary. While there are other approaches to treat water ab initio, the method we have chosen provides sufficient precision for our purposes.
